# Leadership and Presenteeism among Scientific Staff: The Role of Accumulation of Work and Time Pressure

**DOI:** 10.3389/fpsyg.2017.01885

**Published:** 2017-10-25

**Authors:** Carolin Dietz, Tabea Scheel

**Affiliations:** ^1^Chair of Work and Organisational Psychology, Leipzig University, Leipzig, Germany; ^2^Department of Work and Organizational Psychology, Europa-Universität Flensburg, Flensburg, Germany

**Keywords:** presenteeism, scientific staff, leadership, job demands, accumulation, moderated mediation

## Abstract

The present study examines the joint roles of leadership and stressors for presenteeism of scientific staff. Leaders may have an impact on employees' health, both directly through interpersonal interactions and by shaping their working conditions. In the field of science, this impact could be special because of the mentoring relationships between the employees (e.g., PhD students) and their supervisors (e.g., professors). Based on the job demands-resources framework (JD-R), we hypothesized that the pressure to be present at the workplace induced by supervisors (supervisorial pressure) is directly related to employees' presenteeism as well as indirectly via perceptions of time pressure. The conservation of resources theory (COR) states that resource loss resulting from having to deal with job demands weakens the resource pool and therefore the capacity to deal with other job demands. Thus, we hypothesized that accumulation of work moderates the relationship between supervisorial pressure and time pressure, such that the relationship is stronger when accumulation of work is high compared to if accumulation of work is low. Cross-sectional data were obtained from 212 PhD students and postdocs of 30 scientific institutions in Germany. Analysis was performed using the SPSS macro PROCESS (Hayes, 2013). Supervisorial pressure was directly associated with higher presenteeism of employees and indirectly through increased time pressure. Moreover, supervisorial pressure and accumulation of work interacted to predict time pressure, but in an unexpected way. The positive relationship between supervisorial pressure and time pressure is stronger when accumulation is low compared to if accumulation of work is high. It seems possible that job stressors do not accumulate but substitute each other. Threshold models might explain the findings. Moreover, specific patterns of interacting job demands for scientific staff should be considered in absence management.

## Introduction

It is important to analyze and limit the effects of job demands on employees' health. This is reflected by the objective of the German Safety and Health at Work Act (ArbSchG, 1996), that is, to secure and protect employees' health in the workplace through occupational health interventions. In October 2013, an amendment of this law added the assessment of psychological stress at work as a duty of the employers in § 5 (ArbSchG, 1996) stressing the importance of psychological risk factors for the health of the employees. Also, the assessment should be based on empirical results (§ 4 ArbSchG, 1996). The provision of these empirical results is an import task of research in occupational psychology. Thus, this study wants to contribute to a better understanding of the relationship between risk factors (job demands) and health behavior (presenteeism) in the field of scientific research. This field is of crucial importance in the rise of information societies. In 2014, 236,000 persons were officially employed as scientific staff in Germany (Konsortium Bundesbericht Wissenschaftlicher Nachwuchs, 2017), underlining that this field is of considerable relevance.

On the one hand, a sample from this field was chosen because the combination of job demands like unique tasks (e.g., writing a doctoral thesis), which cannot be done by colleagues in a case of absence (lack of replaceability), accumulation of work and time pressure may be very specific. On the other hand, the interactions of different types of job demands are not well understood (van Woerkom et al., 2016), especially in terms of relationships to health behavior like presenteeism (working while being ill). Therefore, the results from other occupations may not be applicable to scientific staff. In the following, we provide an overview of job characteristics that are typical for the scientific field.

The working conditions of scientific staff in different countries are already well described, including the scientific staff in Germany (Cyranoski et al., 2011). For reasons of comparability of the job characteristics, we will focus on young scientific professionals and exclude professors from the analyses. The majority of these young scientific professionals may aim for a PhD (PhD students) or may already have finished their doctor thesis but has no professorial job (postdocs). We will analyze PhD students and postdocs in a single group because their job characteristics may be similar (Konsortium Bundesbericht Wissenschaftlicher Nachwuchs, 2017). For instance, German PhD students and postdocs are often confronted with role conflicts and inconsistent expectations regarding participation in research, teaching, and administration as well as increasing workload and working hours (Kaba-Schönstein and Bonse-Rohmann, 2011; Konsortium Bundesbericht Wissenschaftlicher Nachwuchs, 2013). In Germany, PhD students and postdocs are frequently present and easily accessible in their workplace. They have a special function for the communication of their working group, that is, the participation in administrative tasks, while their integration in the distribution of tasks, responsibilities, and decisions is often unclear (Stock et al., 2002).

Furthermore, Germany also lacks permanently employed, independent instructors and scientists who are employed at a level below full professorships. Codes of conduct in teaching and science bind the majority of PhD students and postdocs while their decision latitude depends on their supervising professors (Borgwardt, 2013). In addition, structural developments and conditions increase the complexity and dynamics within German scientific institutions (Klinder and Fuhrmann, 2011).

In practice, the increasing demands on scientific staff (employed below the level of full professorship) present themselves as increasing evaluations and competitions like teacher evaluations, a rising pressure to publish, and a heightened proportion of research financed by third party funding (Kaba-Schönstein and Bonse-Rohmann, 2011). This as well as the constant need to reapply for jobs because of non-permanent contracts results in very high quantitative and qualitative job demands and consequently in time pressure (Semmer et al., 1999) and accumulation of work, which describes the degree to which work is left undone, for example, in the case of employees' absence through sickness.

However, the exclusive description of the status quo is not sufficient. High job demands like temporary employment have the potential to negatively influence employees' health (e.g., Waenerlund et al., 2011). At the same time, health-related behavior is a mediator between working conditions and health (e.g., Deery et al., 2014). Thus, it is necessary to analyze the relationship between working conditions and health-related behavior; in this case the health-related behavior of academic employees, to further investigate and quantify this negative effects on health in a specific field.

Presenteeism (a health-related behavior) is a serious issue in the workplace. A representative study in Germany showed that fifty percent of the employees worked at least two times within the past 12 months while being ill. Thirty-six percent did this even against medical advice at least once within the past 12 months (DGB-Index Gute Arbeit GmbH, 2009). Moreover, presenteeism has negative correlations with monetary and health outcomes like productivity and decreased future health (and therefore future absence from work; Taloyan et al., 2012; Janssens et al., 2013; Miraglia and Johns, 2015). At the same time, the pressure to be present at the workplace induced by supervisors is positively related to presenteeism in employees even if absence would be legitimate, for example, because of serious illness. This pressure is primarily high in organizations with low replaceability (e.g., in scientific organizations; Marr, 1996; Ashby and Mahdon, 2010; Henneberger and Gämperli, 2014). Also, time pressure and accumulation of work are probable in the context of low replaceability and are positively correlated with presenteeism (Aronsson and Gustafsson, 2005; Demerouti et al., 2009). These direct links seem to point out to mediating mechanisms explaining the relationship between job demands triggering attendance pressure (e.g., supervisorial pressure) and presenteeism (Miraglia and Johns, 2015). However, the understanding of these mediating mechanisms (Miraglia and Johns, 2015) and of the interactions between different job demands (van Woerkom et al., 2016) as well as their relationship with presenteeism is limited, especially in the scientific field.

Therefore, the first aim of this study is to examine the relationships between specific job demands (supervisorial pressure, time pressure, and accumulation of work) and the health behavior of academic staff in case of illness, specifically in the form of presenteeism. A second aim is to contribute to the literature by clarifying how these different job demands interact and how these interactions may be related to presenteeism in the scientific field. The results may contribute to both, future presenteeism research as well as the development of guidelines for practitioners.

**Figure 1:**
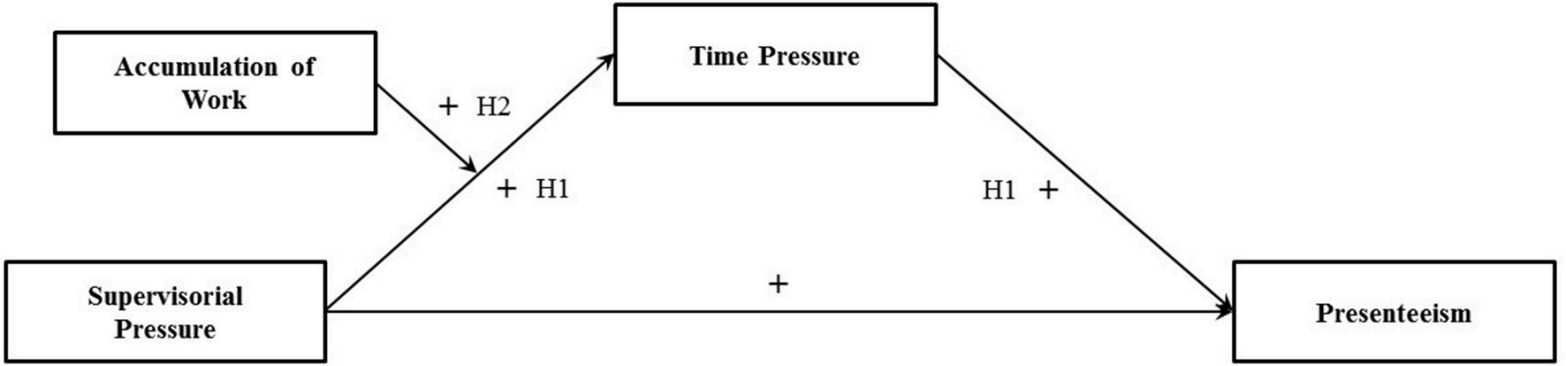
The conceptual model for presenteeism, including the respective hypotheses.

We will first introduce a behavior based concept of presenteeism. Subsequently, antecedents of presenteeism within the context of the job demands-resources framework (JD-R; Bakker et al., 2014) and the conservation of resources theory (COR; Hobfoll, 2001) will be presented. Finally, we will develop our conceptual model (Figure 1) as well as our hypotheses. Our conceptual model states a positive direct and indirect relationship (via time pressure) between supervisorial pressure and presenteeism as well as a moderation of the relationship between supervisorial pressure and time pressure through accumulation of work.

## Presenteeism

Many different definitions of presenteeism are used in the literature. For example, presenteeism is defined as excellent attendance, working with productivity loss, or as the opposite of absenteeism, which is the absence from work (for an overview see Johns, 2010). However, a behavior based definition of presenteeism (Hägerbäumer, 2011) may be preferable (Johns, 2010), for instance, “Presenteeism is the behavior of employees to work while having symptoms of a disease” (Hägerbäumer, 2011, p. 76).

This behavior based definition by Hägerbäumer (2011) provides some advantages. The first advantage is that it is a completely behavior based concept that does neither include any antecedents (e.g., job insecurity) nor consequences (e.g., productivity loss) of presenteeism. Thus, antecedents and consequences can be analyzed empirically and independently from presenteeism, which is important for the scientific utility (Johns, 2010).

The second advantage is that the definition by Hägerbäumer (2011) does not include an evaluation of the behavior like “Sickness presence, that is, going to work despite judging one's current state of health as such that sick leave should be taken” (Aronsson and Gustafsson, 2005, p. 958). This is important because presenteeism can have positive or negative consequences. On the one hand, there may be positive consequences of presenteeism, for instance, on psychological diseases, chronic pain, and musculoskeletal disorders (Bödeker and Hüsing, 2008; Howard et al., 2009). On the other hand, research found an increasing effect of presenteeism on future suboptimal health and sickness absences (Gustafsson and Marklund, 2011; Taloyan et al., 2012).

A case of illness can be seen as a loss of resources and therefore as a stressful situation (Hobfoll, 1989) requiring action (coping strategy) to avoid further loss (e.g., due to missing deadlines). A possible coping strategy may be presenteeism as an attempt to maintain performance and limit detrimental effects due to illness at work. Health status and performance also vary because of present demands (stressors) and available resources. If the employee is not fully recovered from recent demands, job demands can turn into job stressors (Meijman and Mulder, 1998). Within the JD-R (Bakker et al., 2014), job demands can trigger a health impairing process (Bakker and Demerouti, 2007). In stressful situations, for instance getting the flu while facing important deadlines at work, people try to keep up their performance (Hockey, 1993) and to avoid further loss of resources, an attempt which requires physical and/or psychological effort (Hobfoll, 1989; Bakker and Demerouti, 2007). According to the COR theory (Hobfoll, 1989) people draw on other resources like their health, wellbeing, or social capital in stressful situations, in order to avoid a net loss of resources (coping). Because coping is always associated with the use of appreciated resources, coping can initiate a vicious circle of loss and negative net investments for persons with already small repertoires of resources. They therefore try to avoid the loss until all resources are used up (Hobfoll, 1989). Thus, a careful consideration between costs (invested resources) and anticipated benefits (maintaining potentially or actually threatened resources) has to take place (Hägerbäumer, 2011). In fact, job demands may be related to organizational outcomes like presenteeism through strain (Bakker and Demerouti, 2007; Demerouti et al., 2009; Thun and Løvseth, 2016) and impaired health (Miraglia and Johns, 2015).

### Presenteeism and job demands in the scientific field

Presenteeism may have negative consequences for the health of employees (Taloyan et al., 2012; Janssens et al., 2013) and organizational outcomes like productivity (Johns, 2010). Thus, it is important to analyze under which conditions employees are more inclined to show presenteeism as a coping strategy. For this purpose, the relationships between presenteeism and important job demands in the scientific field, namely supervisorial pressure, accumulation of work, and time pressure, were analyzed (conceptual model in Figure 1).

#### Supervisorial pressure

Coping (e.g., presenteeism) takes place in a social and cultural context and interacts with it (Hobfoll et al., 1994; Buchwald and Hobfoll, 2013). Thus, PhD students and postdocs may take into account the behavior of their leader when deciding to come to work or stay at home in case of illness. These decisions could be specific for the scientific field, because of the supervisory mentoring relationship between the leader (e.g., professor) and the PhD students and postdocs.

Leaders in science may be not only supervisors but mentors to the PhD students and postdocs. Relationships of mentors and mentees can be close personal relationships providing vocational and psycho-social support (Scandura, 1998) and thus, are positively correlated with personal and career-related outcomes for both parties like reduced psychological strain and increased scholarly performance (Green and Bauer, 1995; Eby et al., 2013). However, if the immediate supervisor is also the mentor (e.g., professors for PhD students), dysfunctional mentoring relationships may be possible through greater control over assignments and assessments of the mentee by the mentor (Scandura, 1998). The decision to work while being ill, for example, has an impact on both, the PhD students and on their professors. The PhD students may have non-permanent jobs and for this reason deadlines to do their doctoral theses. Also, nobody else can write the doctoral theses. Thus, work would accumulate if the PhD students stay at home while being ill and time pressure would increase. Moreover, the professors may have an interest in successful joint publications with the PhD students to enhance their standing. Explicit (e.g., through a serious conversation) or implicit (e.g., nonverbal signs of dislike) pressure from the professors to be present at the workplace could arise. Such dysfunctional relationships (including supervisorial pressure) can result in higher levels of stress (Feldman, 1999) and increased presenteeism in employees (of a medical insurance business) even if absence was legitimate, for example, because of a serious disease (Ashby and Mahdon, 2010). This pressure is primarily high in organizations with low replaceability (e.g., in scientific organizations; Marr, 1996; Henneberger and Gämperli, 2014). Therefore, we assume that supervisorial pressure is positively related to presenteeism.

#### Time pressure

Time pressure is one of the most stated reasons for presenteeism (Aronsson and Gustafsson, 2005; Hansen and Andersen, 2009; Henneberger and Gämperli, 2014). Also, time pressure is a serious job demand in the scientific field (Semmer et al., 1999). It can be a result of quantitatively and qualitatively very high working demands and appears in the form of a very high workload and/or pace of work (Semmer et al., 1999). High quantitative job demands (time pressure, among others) are related to health indicators like fatigue and headache (Nixon et al., 2011) as well as health behavior like absenteeism (Bakker et al., 2003) and presenteeism (Demerouti et al., 2009).

In addition, we assume that time pressure can explain the link between leadership and employees' health (behavior). This is theoretically supported by the JD-R model (Bakker and Demerouti, 2007), which states that employees' health is affected by provided job resources (e.g., job control) and imposed job demands (e.g., workload; Bakker et al., 2003, 2005). Job demands can trigger a health impairing and therefore presenteeism provoking process via job strain (Demerouti et al., 2009; Miraglia and Johns, 2015) and changed perceptions of the working environment (Zapf et al., 1996), whereas job resources may have motivational character (Bakker and Demerouti, 2007). Indeed, positive supervision can positively influence employees' well-being and task performance as well as negatively influence employees' depression via enhanced (perceived) work characteristics like role clarity, autonomy (partial mediations; Picocolo and Colquitt, 2006; Nielsen et al., 2008), and decreased job insecurity (full mediation; Rigotti et al., 2014). Negative supervision may be positively related to stress and negative affectivity as well as negatively with well-being (Schyns and Schilling, 2013), which can be explained by increased emotional demands (partial mediation; Holstad, 2014) and decreased autonomy (partial mediation; Rooney et al., 2009). Thus, the link between supervision and employees' health can be (partially) explained by (perceived) work characteristics, which could include time pressure. Although Rigotti et al. (2014) found no effect of leadership styles on workload after controlling for the workload baseline, we assume that supervisorial pressure is positively related to (perceived) time pressure. In addition, we expect a remaining positive relationship between supervisorial pressure and presenteeism because leadership can be directly related to employees' health (Schyns and Schilling, 2013) even if mediators are considered (Rigotti et al., 2014). In summary, supervisorial pressure may be partially associated with presenteeism of PhD students and postdocs via time pressure (Figure 1).

*Hypothesis 1*: Time pressure partially mediates the positive relationship between supervisorial pressure and presenteeism.

#### Accumulation of work

As described above, the accumulation of work is an important issue in the scientific field and can be seen as a job demand which may be positively related to presenteeism. Henneberger and Gämperli (2014) postulate that employees are afraid of accumulation of work through absence and accordingly it is a frequently stated reason for presenteeism. The work of PhD students and postdocs (e.g., dissertations, publications) is highly specific. For this reason, the academic field has a lack of replaceability and a high risk of accumulation of work while being absent (e.g., because of illness). For example, Johns (2010) stated that replaceability is a decisive factor for the accumulation of work and thus an important resource within the context of a high workload. However, Böckerman and Laukkanen (2009) found no relationship between replaceability and presenteeism. Aronsson and Gustafsson (2005) showed that employees have low presenteeism scores if the proportion they must take up again on return is small. Primarily organizations with lean structures and difficulties to find agency at short notice (e.g., scientific organizations) have a lack of this resource (replaceability). The inconsistent results may be further explained by additional circumstances like time pressure or workload.

In the context of a lack of replaceability or staff shortage, accumulation of work may relate to time pressure. The more work is left undone (e.g., through absence), the more work has to be taken up and time pressure arises. To our knowledge there are no studies showing this relationship in the academic field, however, there are studies with nurses. Papastavrou et al. (2014) found that the workload of nurses is one of the reasons for rationing (work left undone). Also, Castner et al. (2014) showed that over one third of the variation in missed nursing care (any aspect of required patient care that is omitted or delayed) can be explained by the unit context, that is, the workload at the unit level related to the amount of missed nursing care. This is in line with Al-Kandari and Thomas (2009), who found a positive correlation between increased workload (frequency of tasks) and incompletion of activities during the shift. Elements of care rationing were, for example, patient ambulation, turning, hygiene, mobility, and development and updating of nursing care plans resulting in, for instance, patient falls, pressure ulcers, and nosocomial infections (Papastavrou et al., 2014). One could argue that these results of rationing lead to even more workload and time pressure as the above mentioned incidents go along with even more caring needs and consequently work accumulation. The findings for medical staff may be transferable to academic staff because of similarities in the working conditions, that is, lack of replaceability and time pressure. In summary, a reciprocal relationship between accumulation of work and time pressure seems to be probable.

It may be possible that job demands interact with each other. The consideration of concurrent demands, which increase the probability for negative outcomes through one specific stressor, is well established in the broader stress literature (Lavee et al., 1987). However, according to van Woerkom et al., 2016) the interaction between different types of job demands and the effect of said interaction is not well analyzed. This would imply an underlying assumption that job demands have additive effects, but an intensified effect is plausible within the context of COR (Hobfoll, 2001), because it states that resource loss resulting from dealing with a job demand weakens the resource pool and therefore the capacity to deal with another job demand (vicious circle). Support is provided by psychophysiological research which shows that accumulating demands strain the capacity to cope with these demands and therefore increase the risk for serious disorders through changes in, for instance, health-related behavior (e.g., exercise patterns; Cohen and Wills, 1985), physical functions like immunosuppression and elevated blood pressure as well as cognitive aspects like cumulative fatigue (Cohen et al., 1986). Concerning cumulative fatigue, a positive relationship between perceived workload and (physical and cognitive) fatigue was shown (Myles, 1985; Byström et al., 2004), providing evidence for changes in perceptions of work characteristics moderated by accumulating demands. Moreover, the relationship between emotional demands and absenteeism strengthened with increasing (perceived) workload (van Woerkom et al., 2016), offering further evidence for an interaction between different job demands.

In summary, supervisorial pressure, time pressure, and accumulation of work may be related to higher presenteeism scores. Leadership and accumulation of work seem to be related to time pressure. The job demands supervisorial pressure and accumulation of work may relate to time pressure not additively but multiplicatively (Figure 1). Therefore, the strength of the predicted mediation (Hypothesis 1) of the relationship between supervisorial pressure and presenteeism (through time pressure) should be conditionally influenced by accumulation of work (first stage moderated mediation). We propose that the indirect effect (Hypothesis 1) is stronger if accumulation of work is high compared to if accumulation of work is low.

*Hypothesis 2*: Accumulation of work moderates the positive relationship between supervisorial pressure and time pressure such that the relationship is stronger if accumulation of work is high compared to if accumulation of work is low.

## Method

### Sample and procedure

Representatives of 30 German universities and research institutions agreed to invite all their PhD students and postdocs via mailing lists to our study. Unfortunately, the exact number of contacted persons was not available. However, 415 PhD students and postdocs followed the invitation and completed the online questionnaire.

Participants who answered less than 90% of the items were excluded from the analysis. Further, participants with missing values within the mediator (time pressure) and moderator (accumulation of work) variables as well as independent (supervisorial pressure) and control variables were removed list wise. The 33 participants who indicated that they had not been sick within the last 6 months were excluded. The final sample size is 212 participants, including 139 PhD students, 70 postdocs and 3 persons who did not indicate their academic degree (127 women and 85 men with ages ranging from 24 to 64 years, *M* = 31.4, *SD* = 6.18).

We used *t*-tests to compare the analyzed sample with the excluded participants. They did not show any difference except that the analyzed sample contained a higher percentage of women [*t*_(338)_ = 2.64] and reported worse health status [*t*_(61)_ = −5.07], more irritation [*t*_(63)_ = −2.93], and higher supervisorial pressure to attend while being ill [*t*_(76)_ = 3.99, all *p* < 0.01] than the excluded participants. This could be explained by the exclusion of 33 participants, who indicated that they had not been sick within the last 6 months.

The online questionnaire was created with the software EFS Survey 10.5 (Questback Gmb, 2015). It comprised a total of 155 items and was completed on average within 30 min.

For all translated items the translation model recommended by Brislin (1986) was used, that is, the source was translated to the target language by a bilingual person and another bilingual person blindly translated back to the source. This process was repeated until there was no difference between the original source and the back translated version anymore.

### Measures

#### Presenteeism

Presenteeism was measured with the 6-item presenteeism scale by Hägerbäumer (2011). The participants received the following instruction: “Please indicate how often you have shown the following behaviors in the last 6 months.” This time frame was chosen over the common time frame of 12 months (Johns, 2010) to minimize recall problems. An item was for example “I worked although my doctor advised me not to do it.” On a 5-point Likert scale ranging from 1 (*never in case of illness*) to 5 (*very often in case of illness*) participants indicated how often they showed the described behavior. Participants also had the possibility to indicate that they had not been sick within the last 6 months. The presenteeism scale had a reliability of α = 0.90. Factor analysis supported one component.

#### Supervisorial pressure

Perceived supervisorial pressure to attend in the case of illness was measured with a German translation of the following statement: “I feel under pressure from senior managers to come into work when I am unwell” (Ashby and Mahdon, 2010). Participants responded on a 7-point Likert scale ranging from 1 (*strongly disagree*) to 7 (*strongly agree*).

#### Time pressure

Time pressure was measured with the *Instrument zur stressbezogenen Tätigkeitsanalyse* [instrument for stress-related job analysis] (ISTA; Semmer et al., 1999). The 5-item subscale comprises questions concerning the pace of work and the workload. For example, an item is “How often do you have to work at a high pace?” Participants indicated their responses on a 5-point Likert scale ranging from 1 (*never/seldom*) to 5 (*very often*). The reliability was α = 0.85.

#### Accumulation of work

Accumulation of work was measured with a translated version of an item used by Aronsson and Gustafsson (2005): “If you are absent from work for up to a week, what proportion of your tasks do you must take up again on your return?” Participants indicated their responses on a 4-point Likert scale ranging from 1 (*none or only a small proportion*) to 4 (*virtually all*).

#### Control variables

We assessed control variables potentially related to presenteeism, that is, sex (inconsistent correlations; dichotomously, 0 = *male*, 1 = *female*) and age (trend toward negative correlation; continuously; Bödeker and Hüsing, 2008; Hansen and Andersen, 2009; Badura et al., 2010), as well as type of employing institution, type of researched subject, academic degree, duration of employment in the occupation and at the current institution, relationship status, number of children below the age of 18, responsibility for the financial status of one's household, difficulty to pay bills, health status, chronic disease, health status compared to other people, and irritation.

Aspects related to presenteeism are the specific sector (Henneberger and Gämperli, 2014), the duration of employment (Jourdain and Vézina, 2014), and the professional status (Henneberger and Gämperli, 2014). Some occupational groups, for instance in the education and in the care and welfare sector (Aronsson et al., 2000) and employees with higher responsibilities (Bierla et al., 2013) show higher rates of presenteeism than others. The combination of high job demands and low resources seems to be a good predictor of presenteeism only in the first ten years under these job characteristics (Jourdain and Vézina, 2014). Therefore, the type of the institution, subject, academic degree, and the duration of employment in the current occupation as well as at the current institution were included as control variables. The type of the employing institution was measured with the item “Please indicate the type of the institution you are working for right now.” Participants chose between the response options *university, university of applied science, research institution*, and *other*. The subject was measured with the item “Please indicate your current subject.” The response options were *science and engineering, humanities and social sciences*, and *jurisprudence*. The academic degree was measured with the dichotomous item “Please indicate your professional status” and the response options *postdoc* (0) and *PhD student* (1). The duration of employment in the current occupation and at the current institution were measured continuously with the items “How many years/months have you worked in your current occupation?” and “How many years/months have you worked at your current institution?” Participants indicated the number of years and months in an open answering format.

Moreover, there is evidence for a positive correlation between presenteeism and financial and family responsibilities and conflicts (Hansen and Andersen, 2009; Johns, 2011; Bierla et al., 2013; Henneberger and Gämperli, 2014). Relationship status was measured with the item “Do you live together with a spouse or a partner?” which could be answered with either *yes* (1) or *no* (0); number of children was measured with the item “How many of your children below the age of 18 live in your household?” The participants answered via a free text field. Responsibility for the financial situation of the family was measured with the item “What percentage of the household income do you have to contribute?” The participants responded on a 4-point Likert scale with the response options s*ingle-earner (100%), main earner (more than 50%), joint earner* (ca. *50%*), and *additional earner (less than 50%)* coded as 1, 2, 3, and 4, respectively. Furthermore, difficulty to pay bills was measured with the item previously used by Aronsson and Gustafsson (2005) “Over the previous 6 months, have you had difficulties in handling ongoing expenses for food, rent, and bills?” Participants responded on a 4-point Likert scale with the response options *never over the last 6 months, a couple of times over the last 6 months, a couple of times over the last 3 months*, and *every month*, coded as 0, 1, 2, and 3, respectively.

Furthermore, presenteeism is clearly positively correlated with the health status (Aronsson and Gustafsson, 2005; Hansen and Andersen, 2009; Henneberger and Gämperli, 2014). Thus, health status, chronical disease, health status compared to other people, and irritation (Mohr et al., 2005) were measured as controls. Health status was measured by the item previously used by Güther (2009) “How do you describe your general health?” Participants responded on a 5-point Likert scale ranging from 1 (*excellent*) to 5 (*bad*). Chronic disease was assessed with the dichotomous item by Güther (2009) “Do you suffer from a chronic disease because of which you need medical advice or take medications frequently, at least once in every three months?” with the response options *yes* (0) and *no* (1). Health status compared to other people was measured with the item by Güther (2009) “If you compare yourself with people of your age and your sex, how would you self-assess your disease susceptibility?” Participants responded on a 4-point Likert scale ranging from 1 (*less susceptible*) to 4 (*much more susceptible*). Finally, irritation was measured with the 8-item irritation scale by Mohr et al. (2005). An item was for example “Even at home I often think of my problems at work.” The participants responded on a 7-point Likert scale ranging from 1 (*strongly disagree*) to 7 (*strongly agree*). The internal consistency of the irritation scale was excellent (α = 0.90). Although factor analysis supported the two components (i.e., cognitive and affective) postulated by Mohr et al. (2005), we used a single irritation score.

#### Analysis

For the examination of the hypotheses the SPSS macro PROCESS and the model template seven (Hayes, 2013) was used. Only control variables with a significant correlation with the dependent variable (i.e., presenteeism) were included in the analyses (Spector and Brannick, 2011). Prior to the regression analysis, all variables except for the dependent variable were standardized as the scales do not have a natural zero point and do have different units of measurement (Cohen et al., 2003). Indirect effects and conditional indirect effects based on bootstrapped confidence intervals were used. If the upper and lower level of the confidence intervals do not include zero, the effects are significant at the 95% significance level.

## Results

Descriptives, reliabilities, and correlations between the variables are presented in Table 1. Four control variables were significantly related to presenteeism (i.e., sex, difficulties to pay bills, health status, irritation) and were thus included in the regression analyses (see Table 1).

**Table 1:** Descriptives, reliabilities and correlations between the study variables.

	***M/f***	***SD/n***	**1**	**2**	**3.a**	**3.b**	**3.c**	**3.d**	**4.a**	**4.b**	**4.c**	**5**	**6**	**7**	**8**	**9**	**10**	**11**	**12**	**13**	**14**	**15**	**16**	**17**	**18**	**19**
**CONTROL VARIABLES**																										
1. Sex																										
Female	59.90%	127	–																							
Male	40.10%	85	–																							
2. Age (Years)	31.41	6.18	0.02	–																						
3. Type of employing Institution																										
3.a Dummy-University	24.5%	52	0.15^*^	−0.07	−																					
3.b Dummy-University of applied Science	0.90%	2	−0.02	0.08	−0.06	−																				
3.c Dummy-Research Institution	72.60%	154	−0.16^*^	0.02	−0.95^**^	−0.16^*^	−																			
3.d Dummy-other	0.90%	2	0.08	0.16^*^	−0.06	−0.01	−0.16^*^	−																		
4. Type of Research Topic																										
4.a Dummy-Science and Engineering	67.00%	142	−0.08	−0.15	−0.59^**^	−0.25^**^	0.66^**^	−0.10	−																	
4.b Dummy-Humanities and social Sciences	12.30%	26	0.11	0.15^*^	0.53^**^	0.20^**^	−0.61^**^	0.10	−0.94^**^	−																
4.c Dummy-Jurisprudence	0.90%	2	−0.03	−0.03	0.20^*^	−0.01	−0.18^*^	−0.01	−0.25^**^	−0.05	−															
5. Academic Degree	−	−	0.07	−0.52^**^	0.17^*^	−0.03	−0.18^*^	0.07	0.06	−0.04	−0.04	−														
PhD Students	65.60%	139																								
*Postdoc*	33.00%	70																								
6. Duration of Employment in the Occupation (Months)	56.85	62.43	0.01	0.85^**^	−0.12	0.02	0.09	0.06	−0.05	0.06	−0.05	−0.52^**^	−													
7. Duration of Employment at the current Institution (Months)	48.72	57.12	0.08	0.76^**^	−0.12	0.02	0.11	0.01	−0.05	0.05	−0.01	−0.38^**^	0.86^**^	−												
8. Health Status	2.50	0.79	0.11	0.20^**^	−0.06	0.00	0.04	0.06	0.04	−0.01	−0.07	−0.01	0.17^*^	0.18^*^	−											
9. Chronic Disease	0.25	0.44	0.19^**^	0.07	−0.03	0.06	0.03	−0.06	0.09	−0.04	−0.07	0.09	0.07	0.12	0.32^**^	−										
10. Health Status compared to other People	1.83	0.72	0.18^*^	−0.03	0.02	0.02	−0.02	−0.05	−0.02	0.04	−0.13	0.02	−0.07	−0.05	0.43^**^	0.20^**^	−									
11. Irritation	3.67	1.30	0.15^*^	0.01	0.02	−0.04	−0.02	0.04	0.01	−0.01	−0.01	0.02	−0.03	−0.04	0.23^**^	0.08	0.08	(0.90)								
12. Relationship Status	−	−	0.01	0.16^*^	0.02	−0.02	−0.04	0.08	−0.05	0.03	−0.01	−0.13	0.14^*^	0.15^*^	−0.01	−0.09	0.01	0.04	−							
Yes	57.10%	121																								
No	42.90%	91																								
13. Number of Children	0.35	0.74	−0.03	0.33^**^	0.00	−0.05	−0.03	0.22^**^	−0.08	0.09	−0.05	−0.27^**^	0.28^**^	0.21^**^	0.03	−0.06	0.04	0.09	0.36^**^	−						
14. Responsibility for the financial Status	2.08	1.09	0.07	0.02	0.09	−0.01	−0.11	0.08	−0.03	0.01	0.00	0.01	0.01	0.02	−0.07	−0.09	−0.05	0.01	0.72^**^	0.16^*^	−					
15. Difficulty to pay bills	1.36	0.81	0.06	0.12	0.08	−0.04	−0.14^*^	0.32^**^	−0.14	0.15	−0.05	0.06	0.03	0.01	0.13	0.08	−0.07	0.30^**^	−0.01	0.10	−0.06	−				
**INDEPENDENT VARIABLE**																										
16. Supervisorial Pressure	2.80	1.80	0.25^**^	0.03	−0.01	−0.07	−0.02	0.23^**^	0.00	0.00	0.01	−0.02	0.04	0.04	0.21^**^	0.04	0.18^**^	0.37^**^	0.12	0.10	0.01	0.15^*^	−			
**MEDIATOR**																										
17. Time Pressure	2.97	0.83	0.12	0.05	−0.08	−0.02	0.05	0.16^*^	0.02	−0.01	−0.07	0.02	0.07	0.05	0.17^*^	−0.02	0.04	0.34^**^	0.14^*^	0.10	0.04	0.11	0.55^**^	(0.85)		
**MODERATOR**																										
18. Accumulation of Work	3.39	1.05	0.20^**^	0.17^*^	−0.03	0.01	0.01	0.06	−0.04	0.03	−0.10	−0.12	0.14^*^	0.07	0.01	−0.01	0.08	0.11	0.03	0.05	0.08	−0.03	0.21^**^	0.32^**^	−	
**DEPENDENT VARIABLE**																										
19. Presenteeism	2.36	1.07	0.14^*^	−0.05	0.00	0.01	−0.02	0.10	−0.09	0.07	0.09	−0.01	−0.04	−0.04	0.19^**^	0.02	0.12	0.42^**^	−0.03	0.06	−0.03	0.16^*^	0.53^**^	0.50^**^	0.13	(0.90)

*N = 170 – 212. Reliabilities are reported in parentheses along the diagonal. Sex: Male (0), Female (1), Academic Degree: Postdoc (0), PhD Student (1), Relationship Status: no (0), yes (1)*.

* p < 0.05;

** *p < 0.01*.

Irritation was the only control variable significantly related to time pressure (*B* = 0.15, *SE* = 0.06, *p* < 0.05; Table 2) and presenteeism (*B* = 0.23, *SE* = 0.07, *p* < 0.01). Sex, difficulties to pay bills, and health status were not significantly related to time pressure nor presenteeism. Supervisorial pressure (*B* = 0.49*, SE* = 0.06, *p* < 0.01) was significantly positively related to time pressure. Both, supervisorial pressure (*B* = 0.32*, SE* = 0.07, *p* < 0.01, direct effect) and time pressure (*B* = 0.28, *SE* = 0.07, *p* < 0.01) were significantly positively related to presenteeism.

**Table 2:** Indirect and conditional indirect effects on presenteeism (through time pressure).

**Predictor**	**Time Pressure *B* (*SE*)**	**Presenteeism *B* (*SE*)**	
Control variables			
Sex	−0.07 (0.06)	−0.00 (0.06)	
Difficulties to pay Bills	0.01 (0.06)	0.02 (0.06)	
Health Status	0.04 (0.06)	0.03 (0.06)	
Irritation	0.15 (0.06)^*^	0.23 (0.07)^**^	
Independent variables			
Supervisorial Pressure (*X*)	0.49 (0.06)^**^	0.32 (0.07)^**^	
Time Pressure (*M*)		0.28 (0.07)^**^	
Accumulation of Work (*W*)	0.16 (0.06)^*^		
*X* × *W*	−0.16 (0.07)^*^		
Constant	0.03 (0.06)	2.36 (0.06)^**^	
*R^2^*	0.38^**^	0.39^**^	
**Bootstrap indirect effect on Presenteeism (through Time Pressure)**	***B*** **(*****SE*****)**	**LL 95% CI**	**UL 95% CI**
Time Pressure	0.14 (0.04)^*^	0.07	0.22
**Conditional indirect effects on Presenteeism (through Time Pressure) at three levels of Accumulation of Work**	***B (SE)***	**LL 95% CI**	**UL 95% CI**
Accumulation of Work			
−I *SD* (−1.00)	0.18 (0.06)^*^	0.09	0.31
*M* (0.00)	0.14 (0.04)^*^	0.07	0.22
+ I *SD* (0.58)^a^	0.11 (0.03)^*^	0.05	0.19
Index of moderated mediation	−0.05 (0.02)^*^	−0.10	−0.01

*N = 212. LL, Lower limit; CI, confidence interval; UL, upper limit. Unstandardized regression coefficients are reported. Bootstrap sample size = 1.000*.

a *Maximum obtained value (one SD above the mean was outside the range)*.

* p < 0.05;

** *p < 0.01*.

In line with Hypothesis 1, time pressure partially mediated the positive relationship between supervisorial pressure and presenteeism (*B* = 0.14*, SE* = 0.04, *p* < 0.05; indirect effect). The model explained 38.5% of the variance. Therefore, Hypothesis 1 was supported.

**Figure 2:**
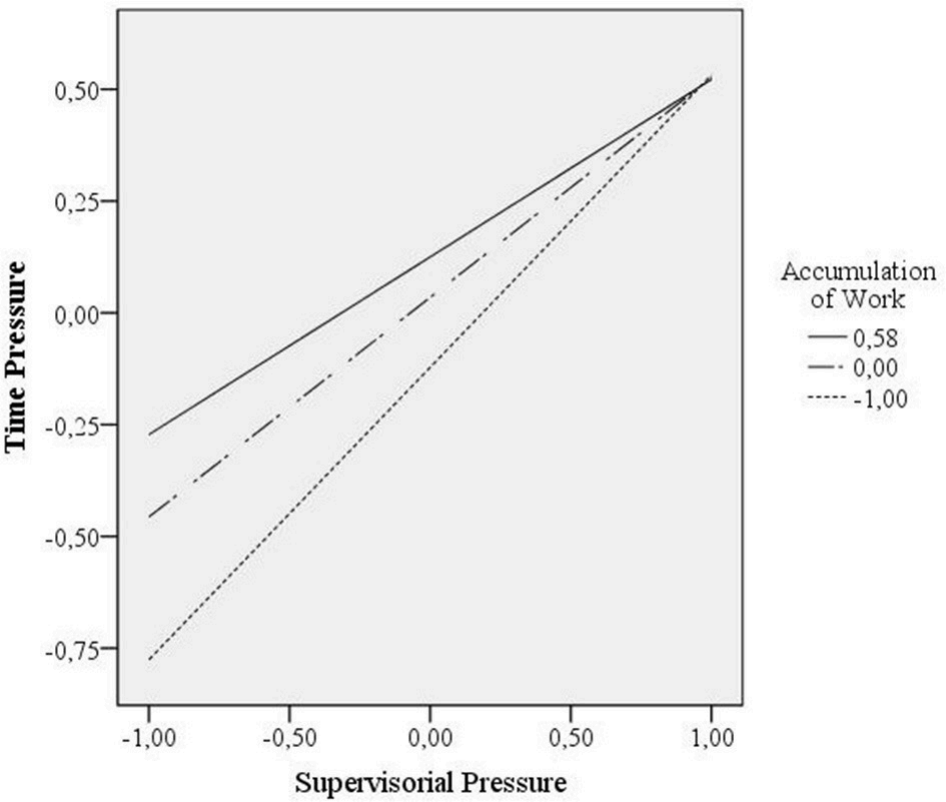
Plot of the two-way interaction effect of Accumulation of Work and Supervisorial Pressure on Time Pressure for three different levels of Accumulation of Work, that is, for the mean, for one *SD* below the mean and for the maximum obtained value (as one *SD* above the mean was outside the range of data).

Hypothesis 2 postulated that accumulation of work moderates the positive relationship between supervisorial pressure and time pressure such that the relationship is stronger if accumulation of work is high compared to if accumulation of work is low (first stage moderated mediation). Accumulation of work (*B* = 0.16, *SE* = 0.06, *p* < 0.05) was significantly positively related to time pressure. Contradicting Hypothesis 2, the interaction between supervisorial pressure and accumulation of work was significantly, but negatively related to time pressure (*B* = −0.16*, SE* = 0.07, *p* < 0.05). The effect of supervisorial pressure on time pressure is weaker if accumulation of work is high (Figure 2).

**Figure 3:**
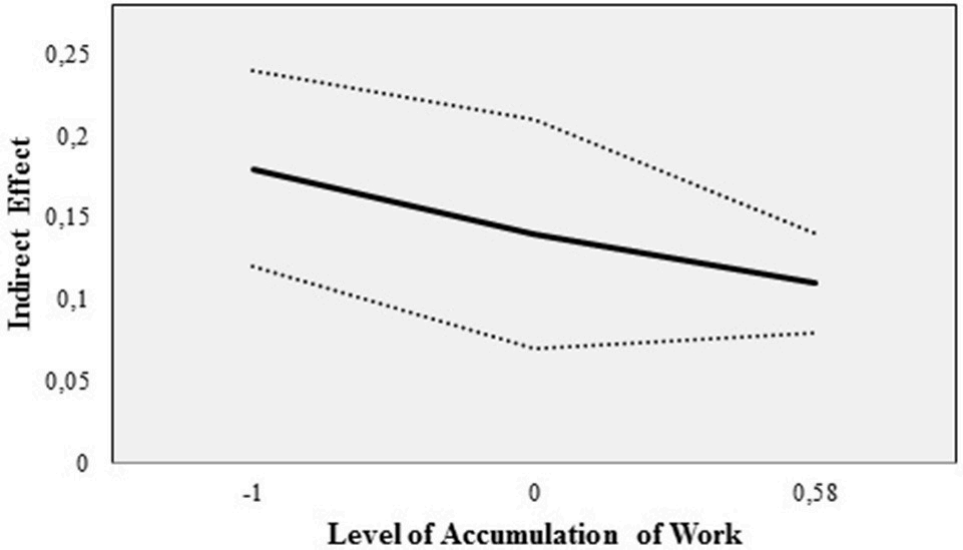
Conditional indirect effect of Supervisorial Pressure on Presenteeism (through Time Pressure) with its continuous lower (−1 *SE*) and upper bounds (+1 *SE*; in dotted lines) at three levels of Accumulation of Work, that is, for the mean, for one *SD* below the mean and for the maximum obtained value (as one *SD* above the mean was outside the range of data).

As additional information, the conditional indirect effect of supervisorial pressure on presenteeism (through time pressure) with its continuous lower (−1 *SE*) and upper bounds (+1 *SE*) are displayed for three different levels of accumulation of work, that is, for the mean, for one *SD* below the mean and for the maximum obtained value (as one *SD* above the mean was outside the range of data; Figure 3).

The conditional indirect effect was positive, significant, and strongest when accumulation of work was low (*B* = 0.18*, SE* = 0.06, *p* < 0.05). For accumulation of work equal to its mean (*B* = 0.14*, SE* = 0.04, *p* < 0.05) and high levels of accumulation (*B* = 0.11*, SE* = 0.03, *p* < 0.05) the conditional indirect effect weakened. The index of moderated mediation was significantly negative (*B* = 0.05*, SE* = 0.02, *p* < 0.05; Table 2). In summary, the data do not support Hypothesis 2, that is, accumulation of work moderates the positive relationship between supervisorial pressure and time pressure such that the relationship is weaker (not stronger) if accumulation of work is high compared to if accumulation of work is low (first stage moderated mediation).

The results of the hypotheses' tests by regression analyses are essentially the same when tested without control variables (respective tables upon request from the authors).

## Discussion

With this study, we contribute to the understanding of psychological risk factors at work in order to help secure and protect employees' health. Job demands can be risk factors because they can lead to negative outcomes, such as increasing health problems (Schaufeli and Taris, 2014). Thus, the first aim of this study was to examine the relationships between specific job demands and the behavior of academic staff in case of illness, that is, presenteeism. The results of this study support that both, supervisorial pressure and time pressure have a positive relationship with presenteeism in the scientific field. This is in line with the findings by Ashby and Mahdon (2010) and Demerouti et al. (2009). Moreover, the data provide evidence for an indirect relationship between supervisorial pressure and presenteeism via time pressure (Hypothesis 1). High levels of supervisorial pressure are related to higher time pressure, which in turn is related to higher levels of presenteeism. These findings are in line with previous findings, which found that supervisors have an influence on employees' (perceived) work characteristics (Picocolo and Colquitt, 2006; Nielsen et al., 2008; Rooney et al., 2009; Holstad, 2014; Rigotti et al., 2014) and that workload is related to health indicators like fatigue and headache (Nixon et al., 2011) as well as health behavior like absenteeism (Bakker et al., 2003) and presenteeism (Demerouti et al., 2009). Thus, this study may support the application of findings about negative supervision, time pressure, and presenteeism from occupations like facility management, banking, auditing, education, and social services to the scientific field.

The second aim of this study was to contribute to the clarification of how different job demands interact and how these interactions may be related to presenteeism in the scientific field. Although well established in the broader stress literature (Lavee et al., 1987), the effect of concurrent demands, which increase the probability for negative outcomes through one specific stressor, is not well researched in professional contexts. Our data provide support for a multiplicative rather than additive interaction of supervisorial pressure and accumulation of work for time pressure. This interplay between the three job demands is related to presenteeism, but in an unexpected way. We argued for a strengthened conditional indirect relationship between supervisorial pressure and presenteeism (via time pressure) if accumulation of work is high. An intensification is plausible within the context of COR (Hobfoll, 2001), because it states that resource loss resulting from dealing with a job demand weakens the resource pool and therefore the capacity to deal with another job demand. Also, van Woerkom et al. (2016) showed for employees of a Dutch mental health care organization that the relationship between one job demand and negative outcomes (absenteeism) strengthened with another increasing work demand. However, our results show that the conditional indirect effect of supervisorial pressure on presenteeism is actually weakened if accumulation of work is high. In other words, in case of high accumulation of work the supervisorial pressure had only a minor association with presenteeism compared to in a situation with low accumulation of work in our study. It seems possible that job demands do not accumulate but substitute each other in the scientific field. Thus, future research is necessary to clarify which findings of interacting job demands in other occupations can be applied to scientific staff. Comparative studies between different occupations may give interesting insights into the complex processes of presenteeism.

Our findings may reflect a substitution effect, that is, substitutes for leadership “will not only tend to affect which leader behaviors (if any) are influential, but will also tend to impact the criterion variable” (Kerr and Jermier, 1978, p. 395). Within the leadership substitutes theory (Kerr and Jermier, 1978), situational (e.g., intrinsically satisfying tasks), individual (e.g., indifference toward rewards), and organizational characteristics (e.g., organizational formalization) can undermine the effect of leadership behavior (Podsakoff et al., 1993; Yagil, 2002). Thus, if the task itself requires frequent attendance, a strict controlling supervisor may be unnecessary. Therefore, accumulation of work may substitute supervisorial pressure and reduce its relationship with time pressure among scientific staff. Also, individual characteristics of PhD students and postdocs, like intrinsic interest in the task, may have an impact. If the task itself is satisfying, supervisorial pressure may be unnecessary to foster the attendance. One indicator may be the low average of supervisorial pressure (*M* = 2.80, *SD* = 1.80, on a seven-point scale). Taking into account the motivational potential of the tasks is a very interesting avenue for future research.

Another interesting issue is the concept of presenteeism for scientific staff itself: supervisors may care less whether their staff attends work though being ill, as long as the work is done anyways. Supervisors may expect their staff to work from home in case of illness, thus presenting a challenge for the notion and measurement of presenteeism in the scientific field but also to the many other kinds of jobs where people may work from home. Accounting for this issue is central to the future research of antecedents and consequences of presenteeism.

Overall, our study contributes to a better understanding of the interplay of leadership, job demands and the behavior in case of illness among scientific staff by considering interactions between different types of job demands and leadership.

Although highly speculative, threshold models following social exchange theories, such as Adams's (1965) equity theory, may explain the results. Adams postulates that people strive for equity, that is, a balanced relationship between their efforts (e.g., working performance) and benefits (e.g., good working conditions) in social contexts. This evaluation includes the comparison of the experienced ratio of efforts and benefits either with a desired reference or with the ratio of significant others. Thereby, equity theory implies the existence of a threshold for reactions toward changes of the ratios. A threshold is passed if the own ratio is significantly different from the ratio of a significant other (e.g., a colleague).

This concept is also known in the literature about psychological contract breach. Rousseau and Tijoriwala (1998) define psychological contracts as “an individual's belief in mutual obligations between that person and another party, such as an employer (either a firm or another person). This belief is predicated on the perception that a promise has been made and a consideration offered in exchange for it, binding the parties to some set of reciprocal obligations” (p. 679). Thus, contract breaches are perceptions by persons that obligations in this exchange process have not been met (Rousseau, 1995). People evaluate if changes in these obligations are in their “zone of acceptance” (Rousseau, 1995, p. 148). If the change is important enough, that is, if the breach is outside the zone of acceptance, people may respond to it, for instance, with quitting their jobs. Although first signs of psychological contract breach do not seem to affect the attitudes of the employees, responses suddenly take place when a threshold is reached (Rigotti, 2009).

It is possible that academic staff evaluates its efforts and benefits ratio in a case of illness. Thus, specific levels of a job demand (e.g., supervisorial pressure) may relate to specific levels of perceived time pressure, that is, to perceived high levels of time pressure and therefore to show high levels of presenteeism while one job demand is increased (Hypothesis 1). Our data may show that high levels of supervisorial pressure are related to highest levels of time pressure, such that a threshold is reached. Thus, if another job demand is increased additionally (e.g., accumulation of work) the strain through these job demands seems to surpass this threshold and the perceived time pressure does not increase anymore (Figure 2). Therefore, the relational pattern between job demands and perceived time pressure might change, namely, the positive relationship between a single job demand and time pressure is weakened.

### Limitations

The main limitation is the cross-sectional design of this study, which does not allow us to deduce causal relationships between the examined variables. For this purpose, longitudinal research designs would be necessary. However, the theoretical model COR and empirical studies support the expected causal direction of the examined relationships. The estimates for the relationships between the variables in this study should be interpreted with caution because of common method bias. However, self-reports may be appropriate to measure the work characteristics in this study as they are operationalized as *perceived* working conditions. Moreover, self-reports of presenteeism should be valid. Presenteeism is a subjective response to a state of feeling unwell while this state may be best assessed by the persons themselves. Therefore, self-reports should theoretically be the best measurement method (Conway and Lance, 2010). We also acknowledge that mediation analysis based on cross-sectional data is not recommended and thus, our findings have a rather exploratory character. The findings call for a longitudinal replication.

The unique sample of scientific staff and accordingly the special supervisory mentoring relationships in the scientific field suggest caution before generalizing our findings to other occupations and fields. However, the role of supervisors beyond the scientific field, for instance, in industrial settings where PhD students are likely to be employed in research and development may include mentoring functions. Within such settings our results may be transferable to occupations with comparable working conditions like high specializations, lack of replaceability and time pressure.

Finally, limitations regarding the measurement have to be considered. Supervisorial pressure and accumulation of work were measured with single items and therefore recall problems, social desirability, and the reliability of the measurement are potential limiting factors. However, these items were used because of economic reasons and for the comparability of the results with the studies by Ashby and Mahdon (2010) as well as Aronsson and Gustafsson (2005).

### Future research

To confirm the findings of this study and to overcome its limitations, more studies are needed addressing the following issues. At first, future studies, especially longitudinal studies should consider indirect effects between different job demands and their potential interactions in explaining presenteeism. Thus, the interplay of job demands and the behavior in case of illness will become more transparent and causal links can be examined.

Secondly, there is a high variety of working conditions for PhD students and postdocs because of factors like different sources of funding, such as scholarships, third party funding, or regular employment at research institutions. Thus, further research should aim to better differentiate within the academic staff and to provide more representative data of PhD students and postdocs.

Another issue relates to gender equality and its legal regulations in different countries, for instance, paid maternal leave. In this study sex has no lasting influence on presenteeism. One reason could be the relatively strong legal protection of parents in Germany and thus, a comparatively smaller amount of additional pressure in a case of pregnancy. However, future research in other legal systems should consider gender issues as sources of attendance pressure.

The fourth issue addresses the measurement of presenteeism. Usually, a single item asking for the recall of presenteeism within a specific time frame is used to measure presenteeism. This can lead to measurement problems like social desirability. In this study, we therefore used a multi-item scale to overcome these problems. Future studies should also use multi-item measurements. The development of such a multi-item measure for English-speaking samples or the translation of the existing German scale by Hägerbäumer (2011) would be desirable. Recall difficulties represent another problem when assessing presenteeism. Therefore, future research might use alternatives to retrospective self-report like diary studies. Also, ever more employees are technically able to work from home, thus calling for a conceptual integration of the issue of “working while ill, at home.”

The fifth issue is related to the highly speculative explanation of the findings with threshold models. In the future, researchers should rethink the existing theories of presenteeism and should develop a more dynamic paradigm to analyze the processes leading to presenteeism.

### Practical implications

The observed relationship between supervisorial pressure to attend and presenteeism are an indicator of the impact of leadership and attendance policies on presenteeism in the academic field. The perceived behavior of supervisors seems to be related to the health behavior of their PhD students and postdocs, both directly and through variables like perceived time pressure. Supervisors should keep this in mind in order to avoid misguided attendance, which might be harmful in the long term (Taloyan et al., 2012; Janssens et al., 2013). Moreover, scientific organizations are already characterized by high specialization of the employees and therefore by a lack of replaceability, time pressure, and accumulation of work. Thus, regarding the findings of this study, additional pressure to attend by supervisors is not useful to increase the attendance of PhD students and postdocs. On the contrary, supervisors should consider their behavior as job resource to enhance the health of their employees and consequently their performance. The effects of supportive leadership on employees' health, like lower back pain (Torp et al., 2001; Elfering et al., 2002), general health, wellbeing, and cases of illness (Kuoppala et al., 2008) as well as presenteeism (Caverley et al., 2007; Miraglia and Johns, 2015) support this.

### Conclusions

This study showed the job demands time pressure and supervisorial pressure to be positively related to presenteeism in the academic field. In addition, supervisorial pressure and presenteeism were indirectly related via time pressure, which conditionally changes for different levels of accumulation of work. Thus, if accumulation of work is high additional supervisorial pressure has only a minor association with presenteeism. The results illustrate the value of rethinking the existing theories of presenteeism, especially in terms of indirect relationships between different job demands and their potential interactions in explaining presenteeism. Furthermore, practitioners should revise attendance policies in the academic field.

## Ethics statement

This study was carried out with informed consent from all subjects about our respect of their confidentiality and anonymity. All subjects participated voluntarily and were free to stop the survey at any time. Based on this assumptions, this kind of study do not need a ethical approval of a committee in Germany.

## Author contributions

Both, TS and CD exchanged research ideas, discussed recent developments of topics like presenteeism and leadership and designed the outline of the article together during several meetings. CD collected data via an online survey within the context of a research project of the Department of Work and Organizational Psychology, Leipzig University, Leipzig (Germany). CD did the preparation and the analyses of the data. Both, TS and CD interpreted and discussed the results during several meetings and colloquia. CD drafted the article and TS revised/edited the article critically for important intellectual content. Both, TS and CD discussed the different versions of the draft during several meetings and telephone calls. TS did the language editing and proofing to improve the clarity of the manuscript and help highlight our research. TS and CD both made substantial contributions to the work reported in the article. Both, TS and CD give their final approval of the version to be published. Both agree to be accountable for all aspects of the work in ensuring that questions related to the accuracy or integrity of any part of the work are appropriately investigated and resolved.CD will act as a corresponding author for the paper.

### Conflict of interest statement

The authors declare that the research was conducted in the absence of any commercial or financial relationships that could be construed as a potential conflict of interest.

## References

[B1] Adams J. S. (1965). Inequity in social exchange, in Advances in Experimental Social Psychology, ed Berkowitz L. (New York; NY: Elsevier), 267–299.

[B2] Al-Kandari F. Thomas D. (2009). Perceived adverse patient outcomes correlated to nurses' workload in medical and surgical wards of selected hospitals in Kuwait. J. Clin. Nurs. 18, 581–590. 10.1111/j.1365-2702.2008.02369.x 19192005

[B3] ArbSchG (1996). Safety and Health at Work Act of 7 August 1996 (Federal Law Gazette I p. 1246), as Last Amended by Article 8 of the Act of 19 October 2013 (Federal Law Gazette I p. 3836).

[B4] Aronsson G. Gustafsson K. (2005). Sickness presenteeism: prevalence, attendance-pressure factors, and an outline of a model for research. J. Occup. Environ. Med. 47, 958–966. 10.1097/01.jom.0000177219.75677.17 16155481

[B5] Aronsson G. Gustafsson K. Dallner M. (2000). Sick but yet at work. An empirical study of sickness presenteeism. J. Epidemiol. Commun. Health 54, 502–509. 10.1136/jech.54.7.502 10846192 PMC1731716

[B6] Ashby K. Mahdon M. (2010). Why Do Employees Come to Work When Ill? An Investigation into Sickness Presence in the Workplace. London: The Work Foundation.

[B7] Badura B. Schröder H. Klose J. Macco K. (eds.). (2010). Fehlzeiten-Report 2009: Arbeit und Psyche: Belastungen Reduzieren - Wohlbefinden Fördern [Absence Report 2009: Work and Psyche: Reducing Stress - Promoting Well-being]. Berlin; Heidelberg: Springer.

[B8] Bakker A. B. Demerouti E. (2007). The job demands-resources model: state of the art. J. Manag. Psychol. 22, 309–328. 10.1108/02683940710733115

[B9] Bakker A. B. Demerouti E. Boer E. de Schaufeli W. B. (2003). Job demands and job resources as predictors of absence duration and frequency. J. Vocat. Behav. 62, 341–356. 10.1016/S0001-8791(02)00030-1

[B10] Bakker A. B. Demerouti E. Euwema M. C. (2005). Job resources buffer the impact of job demands on burnout. J. Occup. Health Psychol. 10, 170–180. 10.1037/1076-8998.10.2.170 15826226

[B11] Bakker A. B. Demerouti E. Sanz-Vergel A. A. (2014). Burnout and work engagement: the JD-R approach. Ann. Rev. Organ. Psychol. Organ. Behav. 1, 389–411. 10.1146/annurev-orgpsych-031413-091235

[B12] Bierla I. Huver B. Richard S. (2013). New evidence on absenteeism and presenteeism. Int. J. Hum. Res. Manag. 24, 1536–1550. 10.1080/09585192.2012.722120

[B13] Böckerman P. Laukkanen E. (2009). What makes you work while you are sick? Evidence from a survey of workers. Eur. J. Public Health 20, 43–46. 10.1093/eurpub/ckp076 19525328

[B14] Bödeker W. Hüsing T. (2008). IGA-REPORT 12 - IGA-Barometer 2. Welle: Einschätzungen der Erwerbsbevölkerung zum Stellenwert der Arbeit, zur Verbreitung und Akzeptanz von betrieblicher Prävention und zur krankheitsbedingten Beeinträchtigung der Arbeit – 2007 [IGA-report 12 – IGA-barometer 2nd wave: evaluation of the value of work, the range and acceptance of occupational prevention and interference with work due to illness with employees - 2007]. Available online at: https://www.google.de/url?sa=t&rct=j&q=&esrc=s&source=web&cd=1&cad=rja&uact=8&ved=0ahUKEwj4_8zcw-nPAhVGqxoKHYFIAQUQFggcMAA&url=http%3A%2F%2Fwww.bds-gewerbevereine.de%2FxFiles%2FContent%2Fcadfd42e-5f9b-4dbc-b6fd-3b449a270333%2FFiles%2Figa_report_12.pdf&usg=AFQjCNEw6iufbrFXfVgbW0QBcm1ZdpqKfg&sig2=Ln_OrB7QMkiaf0DXeCavgg&bvm=bv.136499718,d.d2s

[B15] Borgwardt A. (2013). Arbeitsplatz Hochschule: Aufstieg oder Sackgasse? [University as Workplace: Chance of Promotion or Dead End? Berlin: Friedrich-Ebert-Stiftung.

[B16] Brislin R. W. (1986). The wording and translation of research instruments, in Field Methods in Cross-Cultural Research, ed Lonner W. J. Berry J. W. (Beverley Hills, CA: Sage), 137–164.

[B17] Buchwald P. Hobfoll S. E. (2013). Die Theorie der Ressourcenerhaltung: Implikationen für den Zusammenhang von Stress und Kultur [Conservation of resources theory: implications of the relation between stress and culture], in Handbuch Stress und Kultur: Interkulturelle und kulturvergleichende Perspektiven [Manual of stress and culture: intercultural and culture comparing perspectives], eds Genkova P. Ringeisen T. Leong F. T. L. (Wiesbaden: Springer Fachmedien), 127–138.

[B18] Byström P. Hanse J. J. Kjellberg A. (2004). Appraised psychological workload, musculoskeletal symptoms, and the mediating effect of fatigue: a structural equation modeling approach. Scand. J. Psychol. 45, 331–341. 10.1111/j.1467-9450.2004.00413.x 15281923

[B19] Castner J. Wu Y. W. Dean-Baar S. (2014). Multi-level model of missed nursing care in the context of hospital merger. Western J. Nurs. Res. 37, 441–461. 10.1177/0193945914535670 24869493

[B20] Caverley N. Cunningham J. B. MacGregor J. N. (2007). Sickness presenteeism, sickness absenteeism, and health following restructuring in a public service organization. J. Manag. Stud. 44, 304–319. 10.1111/j.1467-6486.2007.00690.x

[B21] Cohen J. Cohen P. West S. G. Aiken L. S. (2003). Applied Multiple Regression-Correlation Analysis for the Behavioral Sciences, 3rd Edn. Hillsdale, NJ: Erlbaum.

[B22] Cohen S. Evans G. W. Stokols D. Krantz D. S. (1986). Behavior, Health, and Environmental Stress. New York, NY: Plenum Press.

[B23] Cohen S. Wills T. A. (1985). Stress, social support, and the buffering hypothesis. Psychol. Bull. 98, 310–357. 3901065

[B24] Cyranoski D. Gilbert N. Ledford H. Nayar A. Yahia M. (2011). The PhD factory: the world is producing more PhDs than ever before. Is it time to stop? Nature 472, 276–279. 10.1037/0033-2909.98.2.310 21512548

[B25] Conway J. M. Lance C. E. (2010). What reviewers should expect from authors regarding common method bias in organizational research. J. Busin. Psychol. 25, 325–334. 10.1007/s10869-010-9181-6

[B26] Deery S. Walsh J. Zatzick C. D. (2014). A moderated mediation analysis of job demands, presenteeism, and absenteeism. J. Occupat. Organ. Psychol. 87, 352–369. 10.1111/joop.12051

[B27] Demerouti E. Le Blanc P. M. Bakker A. B. Schaufeli W. B. Hox J. (2009). Present but sick: a three-wave study on job demands, presenteeism and burnout. Career Develop. Int. 14, 50–68. 10.1108/13620430910933574

[B28] DGB-Index Gute Arbeit GmbH (2009). DGB Index Gute Arbeit: Der Report 2009 - Wie die Beschäftigten die Arbeitswelt in Deutschland Beurteilen [DGB index good work: the report 2009 – evaluation of work in Germany with employees]. Hamburg: Alsterpaper Available online at: http://index-gute-arbeit.dgb.de/veroeffentlichungen/jahresreports/++co++279bfa3c-dec9-11e3-8bb1-52540023ef1a

[B29] Eby L. T. Allen T. D. Hoffman B. J. Baranik L. E. Sauer J. B. Baldwin S.. (2013). An interdisciplinary meta-analysis of the potential antecedents, correlates, and consequences of protege perceptions of mentoring. Psychol. Bull. 139, 441–476. 10.1037/a0029279 22800296

[B30] Elfering A. Semmer N. K. Schade V. Grund S. Boos N. (2002). Supportive colleague, unsupportive supervisor: the role of provider-specific constellations of social support at work in the development of low back pain. J. Occupat. Health Psychol. 7, 130–140. 10.1037/1076-8998.7.2.130 12003365

[B31] Feldman D. C. (1999). Toxic mentors or toxic proteges?: a critical re-examination of dysfunctional mentoring. Hum. Res. Manag. Rev. 9, 247–278. 10.1016/S1053-4822(99)00021-2

[B32] Green S. G. Bauer T. N. (1995). Supervisory mentoring by advisers: relationships with doctoral student potential, productivity, and commitment. Person. Psychol. 48, 537–562. 10.1111/j.1744-6570.1995.tb01769.x

[B34] Gustafsson K. Marklund S. (2011). Consequences of sickness presence and sickness absence on health and work ability: a Swedish prospective cohort study. Int. J. Occup. Med. Environ. Health. 24, 153–165. 10.2478/s13382-011-0013-3 21526385

[B33] Güther B. (2009). Gesundheitsmonitor: Feld- und Methodenbericht - Welle 15 - Bevölkerungsbefragung/Versichertenstichprobe [Health Monitor: Field and Method Report – Wave 15 – Population Survey/Sample of Insured]. München Available online at: http://gesundheitsmonitor.de/uploads/tx_itao_download/Fragebogen_Welle_15.pdf

[B35] Hägerbäumer M. (2011). Ursachen und Folgen des Arbeitens trotz Krankheit – Implikationen des Präsentismus für das Betriebliche Fehlzeiten- und Gesundheitsmanagement [Antecedents and Consequences of Work While Being ill – Implications of Presenteeism for the Occupational Absence and Health Management]. Retrieved from repOSitorium Univeristy of Osnabrück.

[B36] Hansen C. D. Andersen J. H. (2009). Going ill to work: what personal circumstances, attitudes and work-related factors are associated with sickness presenteeism? Soc. Sci. Med. 67, 956–964. 10.1016/j.socscimed.2008.05.022 18571821

[B37] Hayes A. F. (2013). Introduction to Mediation, Moderation, and Conditional Process Analysis: A Regression-Based Approach. New York, NY: Guilford Press.

[B38] Henneberger F. Gämperli M. (2014). Präsentismus: Ein kurzer Überblick über die ökonomische Relevanz eines Verbreiteten Phänomens [Presenteeism: A Overview of the Economic Relevance of a Widespread Phenomenon]. St. Gallen: Forschungsinstitut für Arbeit und Arbeitsrecht.

[B39] Hobfoll S. E. (1989). Conservation of Resources: a new attempt at conceptualizing stress. Am. Psychol. 44, 513–524. 10.1037/0003-066X.44.3.513 2648906

[B40] Hobfoll S. E. (2001). The influence of culture, community, and the nested-self in the stress process: advancing conservation of resources theory. Appl. Psychol. 50, 337–421. 10.1111/1464-0597.00062

[B41] Hobfoll S. E. Dunahoo C. L. Ben-Porath Y. Monnier J. (1994). Gender and coping: the dual-axis model of coping. Am. J. Commun. Psychol. 22, 49–82. 10.1007/BF02506817 7942644

[B42] Hockey G. R. J. (1993). Cognitive-energetical control mechanisms in the management of work demands and psychological health, in Attention: Selection, Awareness, and Control, eds Baddely A. Weiskrantz L. (Oxford: Clarendon Press), 328–345.

[B43] Holstad T. (2014). The Relation between Leadership and Wellbeing: How Leaders Promote and Undermine Follower Wellbeing. Unpublished Doctorial Dissertation, University Leipzig, Leipzig.

[B44] Howard K. J. Mayer T. G. Gatchel R. J. (2009). Effects of presenteeism in chronic occupational musculoskeletal disorders: stay at work is validated. J. Occupat. Environ. Med. 51, 724–731. 10.1097/JOM.0b013e3181a297b5 19430314

[B45] Janssens H. Clays E. De Clercq B. De Bacquer D. Braeckman L. (2013). The relation between presenteeism and different types of future sickness absence. J. Occupat. Health 55, 132–141. 10.1539/joh.12-0164-OA 23485571

[B46] Johns G. (2010). Presenteeism in the workplace: a review and research agenda. J. Organ. Behav. 31, 519–542. 10.1002/job.630

[B47] Johns G. (2011). Attendance dynamics at work: the antecedents and correlates of presenteeism, absenteeism, and productivity loss. J. Occup. Health Psychol. 16, 483–500. 10.1037/a0025153 21875212

[B48] Jourdain G. Vézina M. (2014). How psychological stress in the workplace influences presenteeism propensity: a test of the Demand–Control–Support model. Euro. J. Work Organ. Psychol. 23, 483–496. 10.1080/1359432X.2012.754573

[B49] Kaba-Schönstein L. Bonse-Rohmann M. (2011). Gesundheitsfördernde Hochschule Esslingen –ein Rahmenkonzept [Health-Promoting University Esslingen – a Conceptual Framework]. Available online at: https://www.hs-esslingen.de/fileadmin/medien/mitarbeiter/jfilipps/08-09-11_Rahmenkonzept_Gesundheitsf%C3%B6rdernde_Hochschule_Kaba-Sch%C3%B6nstein_Bonse-Rohmann.pdf

[B50] Kerr S. Jermier J. M. (1978). Substitutes for leadership: their meaning and measurement. Organ. Behav. Hum. Perform. 22, 375–403. 10.1016/0030-5073(78)90023-5

[B51] Klinder J. Fuhrmann D. R. (2011). Wer kümmert sich um uns und unsere Prozesse?: Zum Stand der Organisationsentwicklung an deutschen Hochschulen - ein Online-Screening (Who takes care of us and our processes?: State-of-the-art organisational development at German universities - an online screening). Wissenschaftsmanagement 2, 16–23.

[B52] Konsortium Bundesbericht Wissenschaftlicher Nachwuchs (2013). Bundesbericht Wissenschaftlicher Nachwuchs 2013. Statistische Daten und Forschungsbefunde zu Promovierenden und Promovierten in Deutschland [Federal Report of Young Scientific Professionals 2013. Statistical Data and Research Findings on PhD Students and Postdocs in Germany]. Bielefeld: Bertelsmann.

[B53] Konsortium Bundesbericht Wissenschaftlicher Nachwuchs (2017). Bundesbericht Wissenschaftlicher Nachwuchs 2017. Statistische Daten und Forschungsbefunde zu Promovierenden und Promovierten in Deutschland [Federal report of young scientific professionals 2017. Statistical data and research findings on PhD students and postdocs in Germany]. Bielefeld: Bertelsmann.

[B54] Kuoppala J. Lamminp,ää A. Liira J. Vainio H. (2008). Leadership, job well-being, and health effects–a systematic review and a meta-analysis. J. Occupat. Environ. Med. Am. College Occup. Environ. Med. 50, 904–915. 10.1097/JOM.0b013e31817e918d 18695449

[B55] Lavee Y. McCubbin H. Olson D. (1987). The effect of stressful life events and transitions on family functioning and well-being. J. Marr. Fam. 49, 857–873. 10.2307/351979

[B56] Marr R. (1996). Absentismus: Der Schleichende Verlust an Wettbewerbspotential. Schriftenreihe Psychologie für das Personalmanagement [Absenteeism: The Creeping Loss of Competitive Potential. Series Psychology for Personnel Management]. Göttingen: Verlag für Angewandte Psychologie.

[B57] Meijman T. F. Mulder G. (1998). Psychological aspects of workload, in Handbook of Work and Organizational Psychology, 2nd Edn., eds Drenth P. J. Thierry H. de Wolff C. J. (Hove: Erlbaum), 5–33.

[B58] Miraglia M. Johns G. (2015). Going to work Ill: a meta-analysis of the correlates of presenteeism and a dual-path model. J. Occup. Health Psychol. 21, 261–283. 10.1037/ocp0000015 26550958

[B59] Mohr G. Rigotti T. Müller A. (2005). Irritation - ein Instrument zur Erfassung psychischer Beanspruchung im Arbeitskontext. Skalen- und Itemparameter aus 15 Studien [Irritation – an instrument assessing mental strain in working contexts. Scale and item parameters from 15 studies]. Zeitschrift für Arbeits und Organisationspsychologie, 49, 44–48. 10.1026/0932-4089.49.1.44

[B60] Myles W. S. (1985). Sleep deprivation, physical fatigue, and the perception of exercise intensity. Med. Sci. Sports Exerc. 17, 580–584. 10.1249/00005768-198510000-00011 4068965

[B61] Nielsen K. Randall R. Yarker J. Brenner S.-O. (2008). The effects of transformational leadership on followers' perceived work characteristics and psychological well-being: a longitudinal study. Work Stress 22, 16–32. 10.1080/02678370801979430

[B62] Nixon A. E. Mazzola J. J. Bauer J. Krueger J. R. Spector P. E. (2011). Can work make you sick?: a meta-analysis of the relationships between job stressors and physical symptoms. Work Stress 25, 1–22. 10.1080/02678373.2011.569175

[B63] Papastavrou E. Andreou P. Efstathiou G. (2014). Rationing of nursing care and nurse–patient outcomes: a systematic review of quantitative studies. Int. J. Health Plan. Manag. 29, 3–25. 10.1002/hpm.2160 23296644

[B64] Picocolo R. F. Colquitt J. A. (2006). Transformational leadership and job behaviors: the medating role of core job charactersitics. Acad. Manag. J. 49, 327–340. 10.5465/AMJ.2006.20786079

[B65] Podsakoff P. M. MacKenzie S. B. Fetter R. (1993). Substitutes for leadership and the management of professionals. Leader. Q. 4, 1–44. 10.1016/1048-9843(93)90002-B

[B66] Questback Gmb H. (2015). EFS Survey [Computer software]. Köln: Questback GmbH.

[B67] Rigotti T. (2009). Enough is enough? Threshold models for the relationship between psychological contract breach and job-related attitudes. Eur. J. Work Organ. Psychol. 18, 442–463. 10.1080/13594320802402039

[B68] Rigotti T. H. Holstad T. Mohr G. Stempel C. H. Hansen E. Loeb C. (2014). Rewarding and Sustainable Healthpromoting Leadership. Dortmund: Bundesanstalt für Arbeitsschutz und Arbeitsmedizin.

[B69] Rooney J. A. Gottlieb B. H. Newby-Clark I. R. (2009). How support-related managerial behaviors influence employees: an integrated model. J. Manag. Psychol. 24, 410–427. 10.1108/02683940910959744

[B70] Rousseau D. M. (1995). Psychological Contracts in Organizations: Understanding Written and Unwritten Agreements. Thousand Oaks, CA: Sage.

[B71] Rousseau D. M. Tijoriwala S. A. (1998). Assessing psychological contracts: issues, alternatives and measures. J. Organ. Behav. 19, 679–695. 10.1002/(SICI)1099-1379(1998)19:1+<679::AID-JOB971>3.0.CO;2-N

[B72] Scandura T. A. (1998). Dysfunctional Mentoring Relationships and Outcomes. J. Manag. 24, 449–467. 10.1177/014920639802400307

[B73] Schaufeli W. B. Taris T. W. (2014). A critical review of the Job Demands-Resources Model: implications for improving work and health, in Bridging Occupational, Organizational and Public Health, eds Bauer G. F. Hämmig O. (Amsterdam: Springer), 43–68.

[B74] Schyns B. Schilling J. (2013). How bad are the effects of bad leaders? A meta-analysis of destructive leadership and its outcomes. Leaders. Q. 24, 138–158. 10.1016/j.leaqua.2012.09.001

[B75] Semmer N. Zapf D. Dunckel H. (1999). Instrument zur stressbezogenen Tätigkeitsanalyse (ISTA) [instrument for stress-related job analysis (ISTA)], in Mensch, Technik, Organisation: Handbuch Psychologischer Arbeitsanalyseverfahren, ed Dunckel H. (Zürich: vdf), 179–204.

[B76] Spector P. E. Brannick M. T. (2011). Methodological urban legends: the misuse of statistical control variables. Organ. Res. Methods 14, 287–305. 10.1177/1094428110369842

[B77] Stock C. Unnold K. Günther-Boehmke G. Meier S. (2002). Die Universität Bielefeld auf dem Weg zu einer gesundheitsfördernden Hochschule [The University Bielefeld on the way to a health-promoting university], in Agenda 21 und Universität - auch eine Frage der Gesundheit, eds Paulus P. Stoltenberg U. (Frankfurt: VAS Verlag), 64–82.

[B78] Taloyan M. Aronsson G. Leineweber C. Hanson L. M. Alexanderson K. Westerlund H. (2012). Sickness presenteeism predicts suboptimal self-rated health and sickness absence: a nationally representative study of the Swedish working population. PLoS ONE 7:44721. 10.1371/journal.pone.0044721 22984547 PMC3439368

[B79] Thun S. Løvseth L. T. (2016). A health impairment hysicians: the mediating role of exhaustion. Health 8, 846–856. 10.4236/health.2016.89089

[B80] Torp S. Riise T. Moen B. E. (2001). The impact of psychosocial work factors on musculoskeletal pain: a prospective study. J. Occupat. Environ. Med. 43, 120–126. 10.1097/00043764-200102000-00010 11227629

[B81] van Woerkom M. Bakker A. B. Nishii L. H. (2016). Accumulative job demands and support for strength use: fine-tuning the job demands-resources model using conservation of resources theory. J. Appl. Psychol. 101, 141–150. 10.1037/apl0000033 26121090

[B82] Waenerlund A.-K. Virtanen P. Hammarström A. (2011). Is temporary employment related to health status? Analysis of the Northern Swedish Cohort. Scand. J. Public Health 39, 533–539. 10.1177/1403494810395821 21321045

[B83] Yagil D. (2002). Substitution of a leader's power bases by contextual variables. Int. J. Organ. Theory Behav. 5, 383–399. 10.1081/OTB-120014897

[B84] Zapf D. Dormann C. Frese M. (1996). Longitudinal studies in organizational stress research: a review of the literature with reference to methodological issues. J. Occup. Health Psychol. 1, 145–169. 10.1037/1076-8998.1.2.145 9547043

